# 10-year trend in quantity and quality of pediatric randomized controlled trials published in mainland China: 2002–2011

**DOI:** 10.1186/1471-2431-13-113

**Published:** 2013-08-02

**Authors:** Chun-Song Yang, Ling-Li Zhang, Li-Nan Zeng, Yi Liang, Lu Han, Yun-Zhu Lin

**Affiliations:** 1 Pharmacy Department, West China Second Hospital, Sichuan University, No. 20, Section 3, Renmin South Road, Cheng du 610041, Sichuan Province, China

**Keywords:** Children, Drugs, Randomized controlled trials, Quality assessment

## Abstract

**Background:**

Quality assessment of pediatric randomized controlled trials (RCTs) in China is limited. The aim of this study was to evaluate the quantitative trends and quality indicators of RCTs published in mainland China over a recent 10-year period.

**Methods:**

We individually searched all 17 available pediatric journals published in China from January 1, 2002 to December 30, 2011 to identify RCTs of drug treatment in participants under the age of 18 years. The quality was evaluated according to the Cochrane quality assessment protocol.

**Results:**

Of 1287 journal issues containing 44398 articles, a total of 2.4% (1077/44398) articles were included in the analysis. The proportion of RCTs increased from 0.28% in 2002 to 0.32% in 2011. Individual sample sizes ranged from 10 to 905 participants (median 81 participants); 2.3% of the RCTs were multiple center trials; 63.9% evaluated Western medicine, 32.5% evaluated traditional Chinese medicine; 15% used an adequate method of random sequence generation; and 10.4% used a quasi-random method for randomization. Only 1% of the RCTs reported adequate allocation concealment and 0.6% reported the method of blinding. The follow-up period was from 7 days to 96 months, with a median of 7.5 months. There was incomplete outcome data reported in 8.3%, of which 4.5% (4/89) used intention-to-treat analysis. Only 0.4% of the included trials used adequate random sequence allocation, concealment and blinding. The articles published from 2007 to 2011 revealed an improvement in the randomization method compared with articles published from 2002 to 2006 (from 2.7% to 23.6%, p = 0.000).

**Conclusions:**

In mainland China, the quantity of RCTs did not increase in the pediatric population, and the general quality was relatively poor. Quality improvements were suboptimal in the later 5 years.

## Background

Randomized controlled trials (RCTs) provide reliable evidence to guide clinical practice. However, difficulties in obtaining a guardian’s consent, obtaining research funding, and ethical concerns [1-3] often limit the conduction of RCTs in children. More attention should be paid to the quality of research in clinical trials evaluating drugs in children, which would lead to improvements in pediatric clinical practices. Al-Namankany et al. [4] showed that the proportion of studies in pediatric dentistry journals using random sequence generation was 28% and allocation concealment was 6%. Crocetti et al. [5] found that a large proportion of pediatric RCT reports in eight prominent journals between July 1, 2007 and June 30, 2008 had a high risk of bias. Hamm et al. [6] also found that more than half of a random sample of 300 pediatric RCTs published in 2007 had a high risk of bias. Wu et al. [7] found that only 6.6% of RCTs published in Chinese journals used adequate methods of random sequence generation. These previous studies provided insightful information on the quality of pediatric RCTs. However, they did not examine whether there was an improvement in quality nor give details of quantitative trends over a period.

In this study, we analyzed RCTs evaluating drug treatment in 17 Chinese pediatric journals in a recent 10-year period to elucidate information about the quantitative trends and quality of these studies in mainland China.

## Methods

We individually searched currently available pediatric journals published from January 1, 2002 to December 30, 2011 in mainland China. There are a total of 17 pediatric medical journals, namely Chinese Pediatrics of Integrated Traditional and Western Medicine, Chinese Journal of Neonatology, Journal of China Pediatric Blood and Cancer, Journal of Clinical Pediatric Surgery, Chinese Journal of Evidence-Based Pediatrics, Journal of Clinical Pediatrics, Journal of Applied Clinical Pediatrics, Chinese Journal of Contemporary Pediatrics, Chinese Journal of Practical Pediatrics, Chinese Journal of Pediatrics, Chinese Journal of Pediatric Surgery, Chinese Journal of Obstetrics & Gynecology and Pediatrics, International Journal of Pediatrics, Chinese Pediatric Emergency Medicine, Journal of Pediatric Pharmacy, Chinese Journal of Perinatal Medicine, and Journal of Pediatrics of Traditional Chinese Medicine.

### Inclusion and exclusion criteria

We included RCTs with participants under the age of 18 years and which used a pharmaceutical intervention. We excluded reviews, self-control studies and trials with more than two intervention groups.

### Selection of studies and data extraction

One reviewer screened the titles and abstracts of every article that was published in the included journals. The full articles were obtained and further evaluated by the selection criteria outlined above. For the selected RCTs, two reviewers independently performed data extraction. The extracted data included: (1) the year of publication; (2) the general characteristics: the research institutions, multicenter or single center, sample size, comparability of baseline characteristics, and the funding resources, etc.; and (3) quality assessment (see Quality assessment section below). Discrepancies were resolved through discussion.

### Quality assessment

Evaluation of the quality of the research methods was based on the Cochrane quality assessment list [8]. Two reviewers independently used the criteria for the evaluation process. The criteria were as follows: (1) random sequence generation; (2) allocation concealment; (3) blinding; (4) incomplete outcome data; (5) selective reporting; and (6) other sources of bias. Discrepancies were resolved through discussion. The details of assessment of bias are listed in Table 1.

**Table 1:** Details of assessment of the risk of bias

	**Allocation sequence**	**Allocation concealment**	**Blinding**	**Incomplete outcome data**	**Selective reporting**	**Other sources of**
	**generation**					**bias**
**Low risk**	The researchers describe a random component in the sequence generation process such as:	Participants and investigators enrolling participants were not aware of assignment.				The study appears to be free of other sources of bias, etc.
	1. A random number table;	1. Central allocation (e.g., telephone/web-based/pharmacy-controlled randomization);	1. No blinding or incomplete blinding, but the review authors judge that the outcome is not likely to be influenced by lack of blinding;	1. No missing outcome data;	1. All outcomes described are included and reported in the analysis;	
	2. Use of a computer random number generator;	2. Sequentially numbered identical drug containers;	2. Blinding of participants and key study personnel ensured, and unlikely that the blinding could have been broken, etc.	2. Missing outcome data balanced in number across intervention groups, with similar reasons for missing data across groups;	2. For registered trials, all outcomes reported are included in the analysis;	
	3. Coin tossing;	3. Sequentially numbered, opaque, sealed envelopes, etc.		3. Missing data have been imputed using appropriate methods, etc.	3. All outcomes expected to have been collected for the condition are reported, etc.	
	4. Throwing dice;					
	5. Drawing of lots, etc.					
**High risk**		Participants or investigators enrolling participants could possibly know the assignment.				
	1. Sequence generated by odd or even date of birth;	1. Use of an open random allocation schedule;	1. No blinding or incomplete blinding for an outcome that was likely to be affected by blinding.	1. Reason for missing outcome data likely to be related to true outcome, with either imbalance in numbers or reasons for missing data across intervention groups;	1. Not all of the study’s pre-specified primary outcomes have been reported;	1. Had a potential source of bias related to the specific study design used;
	2. Rule based on date (or day) of admission;	2. Date of birth;	2. Blinding procedures could have been broken, etc.	2. “As-treated” analysis done with substantial difference in the intervention received from that assigned at randomization, etc.	2. One or more primary outcomes is reported using measurements, analysis methods or subsets of the data (e.g., subscales) that were not pre-specified;	2. Has been claimed to have been fraudulent, etc.
	3. Based on hospital or clinic record number, etc.	3. Case record number, etc.			3. The study report fails to include results for a key outcome that would be expected to have been reported for such a study, etc.	
**Unclear**	Randomization not described.	Insufficient evidence to permit judgment, etc.	Insufficient information to permit judgment;	1. Insufficient reporting of attrition/exclusions to permit judgment;	Insufficient information for clear decision, etc.	Insufficient information for assessment.
			The study did not address this outcome, etc.	2. The study did not address this outcome, etc.		

### Data management and analysis

Continuous variables were analyzed using mean values and standard deviations or median and interquartile range, while categorical variables were analyzed using percentages. The Student *t*-test and Mann–Whitney *U* test were used to test differences in continuous variables as appropriate, and the *χ*^2^ test was used for proportions. Data management and analysis were performed with the SPSS v. 16.0 (SPSS Inc., Chicago, IL, USA).

## Results

### General characteristics

**Figure 1:**
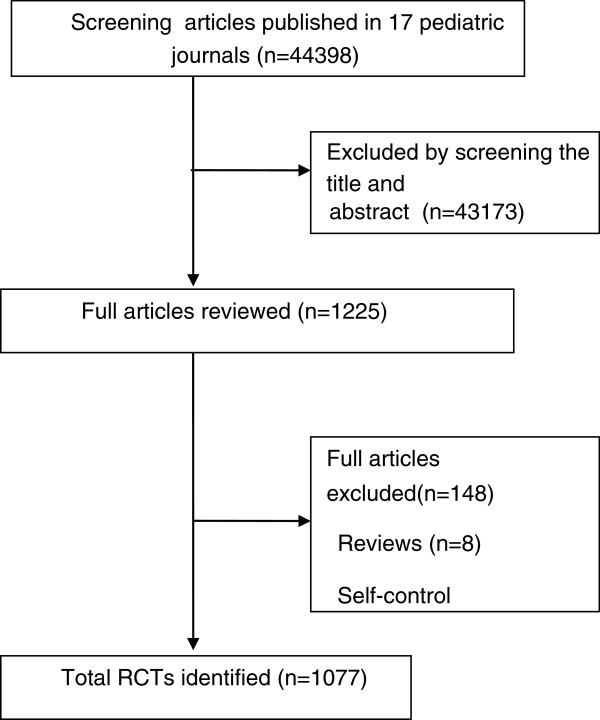
Flow diagram showing selection of the trials.

**Figure 2:**
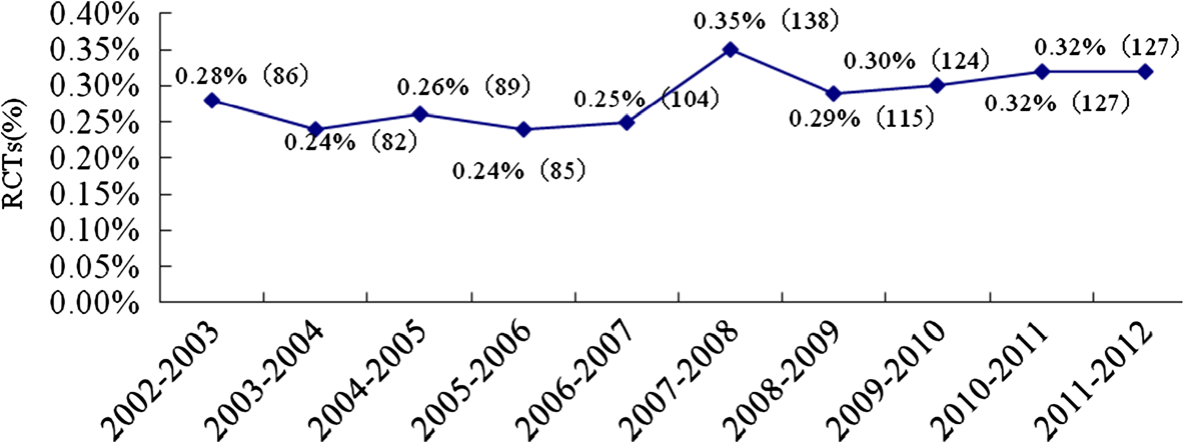
Increase in the quantity of pediatric RCTs published in mainland China from 2002 to 2011.

**Figure 3:**
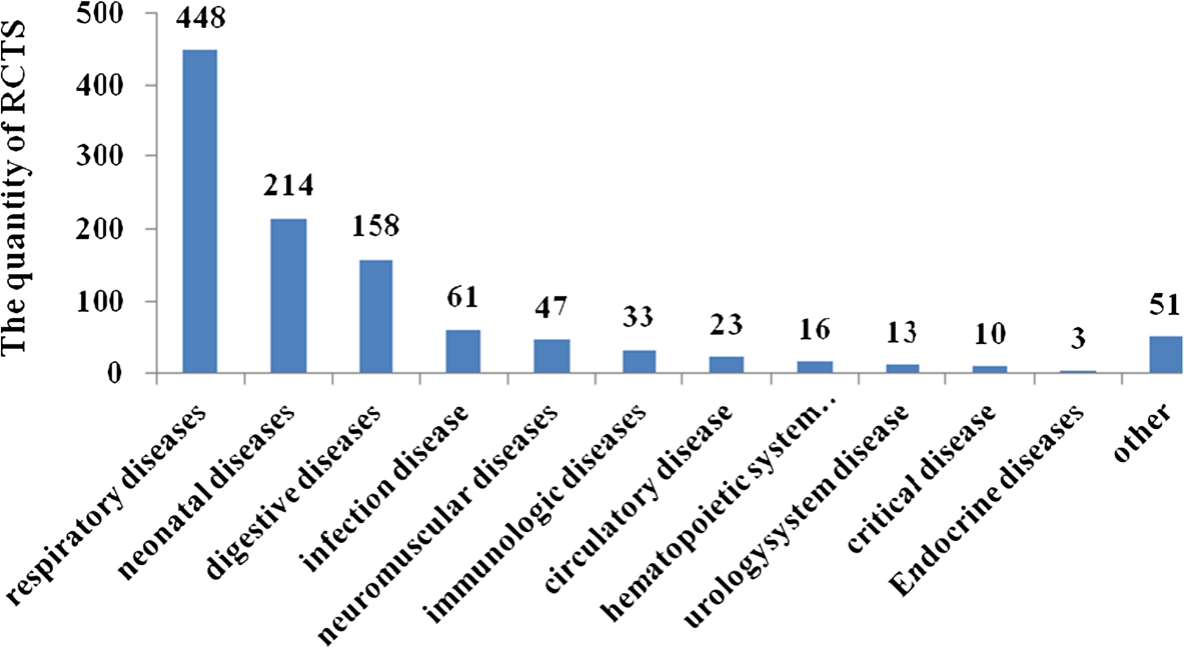
Distribution of disease states among 1077 RCTs in pediatric journals published in mainland China from 2002 to 2011.

After screening 44398 articles published in the 17 Chinese pediatric medical journals, a total of 1077 RCTs were included in the analysis (Figure 1). Over the 10-year period, the proportion of RCTs in mainland China increased from 0.28% in 2002 to 0.32% by 2011 (Figure 2). There was no significant increase in the number of RCTs conducted annually. Only 4.09% of the trials reported financial funding; however, we could not confirm that how many trials were sponsored by pharmaceutical companies because the information was not given. Individual study sample sizes ranged from 10 to 905 (median 81 participants). Only 0.6% (7/1077) of RCTs reported that calculation of sample size had been performed, and 2.3% (25/1077) of RCTs included multiple centers, with the number of participating centers ranging between two and ten. Nearly one-quarter (254/1077) of RCTs were conducted in teaching hospitals, 63.9% (688/1077) evaluated Western medicine, 32.5% (350/1077) evaluated traditional Chinese medicine, and 3.6% (39/1077) evaluated a combination of both traditional Chinese and Western medicine (Table 2). The top five categories of disease included respiratory (41.6%), neonatal (19.9%), digestive (14.7%), infection (5.7%), and neuromuscular (4.4%) diseases (Figure 3).

**Table 2:** General characteristics of the included pediatric RCTs

**Items**	**Quantity**
	**(n, %)**
**Research institute**	
Teaching hospitals	254 (23.6%)
Non-teaching hospitals	818 (75.9%)
Scientific research institutions	5 (0.5%)
**Study center**	
Multiple	25 (2.3%)
Single	1052 (97.7%)
**Funding**	
Not stated	1033 (95.91%)
Stated	44 (4.09%)
International	1 (0.09%)
National	9 (0.84%)
Provincial	17 (1.58%)
Municipal	14 (1.3%)
University	3 (0.28%)
Pharmaceutical company sponsored	Unclear
**Intervention**	
Western medicine	688 (63.9%)
Traditional Chinese medicine	350 (32.5%)
Combined traditional Chinese and Western medicine	39 (3.6%)
**Comparability of baseline**	
Comparable	936 (86.9%)
Incomparable	2 (0.2%)
Unclear	139 (12.9%)
**Control intervention**	
Placebo control	12 (1.1%)
Positive drug	461 (42.8%)
Open control	604 (56.1%)
**Sample size calculation**	
Stated	7 (0.6%)
Not stated	1070 (99.4%)
**Outcomes**	
Positive	1028 (95.5%)
Negative	49 (4.5%)
**Report of adverse events**	
Stated adverse events	279 (25.9%)
Stated no adverse events	251 (23.3%)
Not stated	547 (50.8%)

### Quality assessment

#### Randomization methods

The results are presented in Table 3: 15% (161/1077) of RCTs used an adequate method of random sequence generation; 74.6% (803/1077) denoted “random allocation” in the study without a specific description; and 10.4% (113/1077) regarded quasi-randomization as correct randomization, and the most common methods for allocating participants used were the visiting sequence of inpatient/outpatient, the card number of the inpatient/outpatient number or patient birth date.

**Table 3:** Reporting of methodological characteristics of pediatric RCTs

**Items**	**Quantity**
	**(n, %)**
**Method of random sequence generation**	
**Low risk**	161 (14.9%)
Random number table	121 (11.2%)
Drawing of lots	28 (2.6%)
Computer	9 (0.8%)
Lottery	1 (0.1%)
Coin toss	1 (0.1%)
Dice	1 (0.1%)
**High risk**	
Visiting sequence of inpatient/outpatient, the card number of inpatient/outpatient number and birth date.	113 (10.5%)
**Unclear**	803 (74.6%)
**Method of allocation concealment**	
**Low risk**	10 (1%)
Central allocation	7 (0.7%)
Sealed opaque envelopes	3 (0.3%)
**High risk**	--
**Unclear**	1067 (99%)
**Blinding**	
**Low risk**	6 (0.6%)
Single blind (participants)	3 (0.3%)
Double blind (participants and investigators)	2 (0.2%)
Triple blind (participants, investigators, outcome assessors)	1 (0.1%)
**High risk**	--
**Unclear**	1071 (99.4%)
**Incomplete outcome data**	
**Low risk**	988 (91.7%)
**High risk**	--
**Unclear**	89 (8.3%)
**Selective reporting**	
**Low risk**	--
**High risk**	18 (1.7%)
**Unclear**	1059 (98.3%)
**Other bias**	
**Low risk**	--
**High risk**	2 (0.2%)
**Unclear**	1075 (99.8%)

-- means no data available.

#### Allocation concealment

Only 1% (10/1077) of RCTs reported reasonable allocation concealment, including central allocation and sealed opaque envelopes; 99% (1067/1077) of trials did not indicate the allocation concealment method.

#### Blinding

Only 0.6% (6/1077) of RCTs reported the blinding method (0.3% single-blind for participants, 0.2% double-blind for participants and investigators, 0.1% triple-blind for participants, investigators and assessors); 1.6% of RCTs (17/1077) reported blinding without full disclosure of the method; and 97.8% (1054/1077) did not mention the use of blinding in the trials.

#### Interventions and outcomes measurement

Only 1.1% (22/1077) of RCTs were placebo-controlled, 56.1% (604/1077) were open control and 42.8% (461/1077) were positive drug control. “Open control” was defined as having two groups with the same standard treatment with intervention in the treatment group (i.e., standard treatment + test drug *versus* standard treatment). “Positive drug control” was defined as the control group having an active control (i.e., test drug *versus* control drug, or standard treatment + test drug *versus* standard treatment + control drug). A positive result was reported in 95.5% (1028/1077) of trials; 4% (43/1077) of trials used laboratory data as indicators of efficacy and 58% (625/1077) used endpoint outcomes, while 38% (409/1077) used both laboratory data and endpoint outcomes. The follow-up period ranged from 7 days to 96 months, with a median of 7.5 months. Incomplete outcome data was reported in 8.3% (89/1077) of trials, of which 4.5% (4/89) used intention-to-treat analysis; 49.2% (530/1077) of trials reported adverse events, of which 52.6% (279/530) reported specific adverse events, and 47.4% (251/530) reported no adverse events; 1.7% (18/1077) of trials showed obvious selective reporting (outcomes described in the methodology were not reported in the results); in 98.3% (1059/1077) the possibility of selective reporting could not be evaluated because none of the included studies had been registered, and we were unable to compare the research with the protocol. In general, 0.4% (4/1077) reported all random sequence, allocation concealment, and blinding methods.

#### Bias from other sources

In 0.2% (2/1077) of RCTs, the reported baseline data in the treatment and control groups were not comparable.

#### Subgroup analysis

We conducted a subgroup analysis to determine which factors influenced the quality of the included studies. The quality of RCTs conducted in multiple centers was superior to those of single centers regarding random sequence generation, allocation concealment, and blinding. The quality of allocation concealment and blinding but not random sequence generation, was also better in teaching hospitals compared with non-teaching hospitals. In contrast, incomplete outcome data was more likely in RCTs from multiple centers and in teaching hospitals. Trials with financial funding performed better for allocation concealment than trials without funding. However, we could not explore the influence of pharmaceutical industry funding on quality because sponsorship information was not reported. The RCTs published from the 2007 to 2011 revealed an improvement in the randomization method compared with 2002–2006 (from 2.7% to 23.6%). There was also a trend for improvement in allocation concealment (p = 0.053) (Table 4).

**Table 4:** Reporting of methodological characteristics for different subgroups

**Item**	**Multiple-center**	**Single-center**	**p**	Teaching hospitals*	**Non-teaching**	**p**	**Funding**	**No funding**	**p**	**2002-2006**	**2007-2011**	**p**
	**(n = 25)**	**(n = 1052)**		**(n = 254)**	hospitals* (n = 818)		**(n = 44)**	**(n = 1033)**		**(n = 446)**	**(n = 631)**	
**Adequate random**	13 (52%)	148 (14.06%)	0.000	46 (18.11%)	114 (13.94%)	0.103	6 (13.64%)	155 (15%)	0.803	12 (2.69%)	149 (23.61%)	0.000
**sequence generation**												
**(n = 161)**												
**Adequate allocation**	5 (20%)	5 (0.48%)	0.000	9 (3.54%)	1 (0.12%)	0.000	3 (6.82%)	7 (0.68%)	0.006	1 (0.22%)	9 (1.43%)	0.053
**concealment**												
**(n = 10)**												
**Adequate blinding**	2 (8%)	4 (0.38%)	0.007	5 (1.97%)	0 (0%)	0.001	1 (2.27%)	5 (0.48%)	0.222	1 (0.22%)	5 (0.79%)	0.410
**(n = 6)**												
**Incomplete outcome**	12 (48%)	976 (92.78%)	0.000	214 (84.25%)	769 (94.01%)	0.000	29 (65.9%)	959 (92.84%)	0.000	416 (93.27%)	572 (90.65%)	0.123
**data (low risk)**												
**(n = 988)**												
**Selective reporting**	0 (0%)	18 (1.71%)	1.000	3 (1.18%)	15 (1.83%)	0.588	0 (0%)	18 (1.74%)	1.000	6 (1.35%)	12 (1.9%)	0.483
**(n = 18)**												

*We excluded five studies conducted in scientific research institutions.

## Discussion

This study showed that the number and the quality of Chinese pediatric RCTs improved slightly during the past decade. It had been reported that the number of adult RCTs was 2.7 times that of pediatric RCTs in six medical journals [9]. The number of pediatric RCTs increased from 0.4 to 16.9 per year, while adult RCTs increased from 4.71 to 90.5 per year [10-13]. Owing to a lack of pediatric RCTs, use of drugs in pediatric settings are often forced to extrapolate from the results of clinical trials in adults, which is inappropriate [10,14]: on the one hand, there are many differences in physiological function, pharmacokinetics, and pharmacodynamics between children and adults, thus the administration of unevaluated pharmaceutics in children by extrapolation could have no effect or be harmful [15]; on the other hand, this would result in off-label drug use, which is relevant to the occurrence of adverse events [16]. Reports indicate that the rate of off-label pharmaceutical use in pediatric settings ranges from 11% to 46% [17-23]. Recently, a survey suggested that the rate of off-label drug use in China is 78.96% in hospitalized children [24]. In accordance with the prevalence of off-label drug use in pediatric settings, the risk of adverse effects has been reported to be three times as high as in adults [25].

There are five major reasons for the limited clinical trials of drugs in children: (1) the spectrum of diseases is narrow and the incidence of disease in children is low compared with adults [11]; (2) difficulties in obtaining consent from guardians limit the number of participants available for clinical trials [3,14,26]; (3) the unwillingness of researchers to conduct research as a result of the extensive pressures exerted by ensuring participants’ rights in trials and minimizing risk in clinical trials [27]; (4) the limited financial support from pharmaceutical companies because of the high cost of research and the low beneficial return; and (5) the lack of public policies supporting a re-evaluation of procedures for off-label drug use [11,15].

There was a median of 81 participants in the published pediatric RCTs in China, which is lower than in international studies (median of 272 participants). In addition, the proportion reporting sample size calculation in Chinese pediatric RCTs was also lower than in international pediatric RCTs (4–65%) [11,28] and adult RCTs (23–64%) [28-30]. The proportion of multiple center pediatric RCTs in China (2.3%) was also lower than the adult RCTs (28–67%) worldwide [11,28].

Inadequate or unclear research methods of allocation concealment and random allocation signify low quality clinical trials and may exaggerate efficacy by as much as 30–41% [31-33]. Compared with the analysis of studies in pediatric dentistry conducted by Al-Namankany et al. [4], the proportion of studies with adequate randomization and blinding was low, while use of intention-to-treat analysis was similar to our study. Compared with adult RCTs, the proportion with adequate randomization in our study was similar to the international level (14–39%), and allocation concealment, blinding, and intention-to-treat analysis were at a lower level than the international standard (allocation concealment 13–40%, blinding 19–45%, intention-to-treat analysis 12–43%) [28-30,34].

The main problems in the pediatric RCTs in China were as follows: (1) studies were often labeled “random” without giving details on random sequence generation, and some researchers erroneously regarded the quasi-random process as the correct method for randomization; (2) outcome measurements using blinding methodology were ignored, and explanations of the use of blinding methodologies were vague; (3) most RCTs used active drugs whose efficacy was unclear as control, and few used a placebo control; (4) RCTs focused on the evaluation of short-term efficacy, and had no discussion of long-term efficacy; (5) adverse effects were often ignored or not reported; and (6) there was lack of clinical trial registration making it difficult to monitor the quality of the conduction and reporting of trials. Further studies should overcome these drawbacks.

The quality of the published RCTs in China did show an increase in the reported random sequence generation methods in studies published from 2007 to 2011 compared with 2002 to 2006. The proportion with adequate use of randomized sequence generation methods increased from 2.7% to 23.6%. The following factors may have contributed to the increased use of randomized sequence generation: (1) a rapid development of evidence-based medicine in China; (2) greater awareness by clinicians of the importance of quality in clinical research for clinical decision-making; and (3) improved quality of research, with Consolidated Standards Of Reporting Trials(CONSORT) publications available to researchers to scrutinize standard RCTs. Compared with the quality of international clinical trials, only 25% of the pediatric trials published in China achieved adequate random sequence generation, which still leaves major opportunities for improvement. The quality of multiple center RCTs in China was better than in single center studies, which was consistent with international reports [35].

There were several limitations in our study: (1) assessment of the quality of RCTs was based on the description in the articles without verification from the original authors. However, a verification process suggested there would be no significant improvement in the assessment [7]; (2) we only included RCTs of drug interventions and excluded non-drug therapy (such as physical therapy or surgery); in addition, we also excluded more than two intervention groups, and as it is more difficult to control quality in these studies, our study may have overestimated the quality of pediatric RCTs in China; and (3) we only included trials published in pediatric professional journals, and other general medical journals could contain pediatric RCTs.

## Conclusions

In mainland China, the quantity of RCTs did not increase over 10 years in the pediatric population, and the general quality remained relatively poor. There was an improvement in quality in the latest 5 years, but this was suboptimal.

## Abbreviations

RCTs: Randomized controlled trials; CONSORT: Consolidated Standards Of Reporting Trials.

## Competing interests

The authors declare that they have no competing interests.

## Authors’ contributions

C-SY: designed the study, selected trials for inclusion, extracted data from. papers, appraised the quality of studies, carried out analysis, interpretation of the data, draft the manuscript, and approved the final manuscript as submitted. L-LZ: designed the study, selected trials for inclusion, appraised the quality of studies, interpretation of the data, draft the manuscript, and approved the final manuscript as submitted. L-NZ: undertook searches, selected trials for inclusion, extracted data from papers and appraised the quality of papers, commented the manuscript, and approved the final manuscript as submitted. YL: undertook searches, selected trials for inclusion, extracted data from papers and appraised the quality of papers, commented the manuscript, and approved the final manuscript as submitted. LH: undertook searches, extracted data from papers, entered data into SPSS and checked the data, commented the manuscript, and approved the final manuscript as submitted. Y-ZL: undertook searches, extracted data from papers, entered data into SPSS, checked the data and commented the manuscript, and approved the final manuscript as submitted.

## Pre-publication history

The pre-publication history for this paper can be accessed here:

http://www.biomedcentral.com/1471-2431/13/113/prepub
